# A synthesis of patterns of environmental mercury inputs, exposure and effects in New York State

**DOI:** 10.1007/s10646-020-02291-4

**Published:** 2020-11-10

**Authors:** D. C. Evers, A. K. Sauer, D. A. Burns, N. S. Fisher, D. C. Bertok, E. M. Adams, M. E. H. Burton, C. T. Driscoll

**Affiliations:** 1 Biodiversity Research Institute, 276 Canco Road, Portland, ME 04103 USA; 2 Syracuse University, Syracuse, NY 13244 USA; 3 U.S. Geological Survey, Troy, NY 12180 USA; 4 School of Marine and Atmospheric Sciences, Stony Brook University, Stony Brook, NY 11794 USA; 5 New York State Energy Research and Development Authority, Albany, NY 12203 USA

**Keywords:** Mercury, Air, Fish, Bird, New York

## Abstract

Mercury (Hg) pollution is an environmental problem that adversely affects human and ecosystem health at local, regional, and global scales—including within New York State. More than two-thirds of the Hg currently released to the environment originates, either directly or indirectly, from human activities. Since the early 1800s, global atmospheric Hg concentrations have increased by three- to eight-fold over natural levels. In the U.S., atmospheric emissions and point-source releases to waterways increased following industrialization into the mid-1980s. Since then, water discharges have largely been curtailed. As a result, Hg emissions, atmospheric concentrations, and deposition over the past few decades have declined across the eastern U.S. Despite these decreases, Hg pollution persists. To inform policy efforts and to advance public understanding, the New York State Energy Research and Development Authority (NYSERDA) sponsored a scientific synthesis of information on Hg in New York State. This effort includes 23 papers focused on Hg in atmospheric deposition, water, fish, and wildlife published in *Ecotoxicology*. New York State experiences Hg contamination largely due to atmospheric deposition. Some landscapes are inherently sensitive to Hg inputs driven by the transport of inorganic Hg to zones of methylation, the conversion of inorganic Hg to methylmercury, and the bioaccumulation and biomagnification along food webs. Mercury concentrations exceed human and ecological risk thresholds in many areas of New York State, particularly the Adirondacks, Catskills, and parts of Long Island. Mercury concentrations in some biota have declined in the Eastern Great Lakes Lowlands and the Northeastern Highlands over the last four decades, concurrent with decreases in water releases and air emissions from regional and U.S. sources. However, widespread changes have not occurred in other ecoregions of New York State. While the timing and magnitude of the response of Hg levels in biota varies, policies expected to further diminish Hg emissions should continue to decrease Hg concentrations in food webs, yielding benefits to the fish, wildlife, and people of New York State. Anticipated improvements in the Hg status of aquatic ecosystems are likely to be greatest for inland surface waters and should be roughly proportional to declines in atmospheric Hg deposition. Efforts that advance recovery from Hg pollution in recent years have yielded significant progress, but Hg remains a pollutant of concern. Indeed, due to this extensive compilation of Hg observations in biota, it appears that the extent and intensity of the contamination on the New York landscape and waterscape is greater than previously recognized. Understanding the extent of Hg contamination and recovery following decreases in atmospheric Hg deposition will require further study, underscoring the need to continue existing monitoring efforts.

## Introduction

Mercury pollution is an environmental problem with local, regional, and global dimensions that adversely affects human and ecosystem health—including that in New York State, USA. Mercury (Hg) is a naturally occurring element that is emitted from volcanoes and released by natural processes such as soil weathering (Driscoll et al. 2013). However, more than two-thirds of the Hg currently released to the environment globally originates, either directly or indirectly, from human activities (Amos et al. 2013). For more than 200 years, Hg has been released into the atmosphere and waterways in New York State by human activities, including emissions from fossil fuel combustion, waste incineration, metal smelting, and chlorine production, and discharges to surface waters from domestic and industrial wastewater. Since the early 1800s, global releases have increased atmospheric Hg concentrations by three- to eight-fold above natural levels (Streets et al. 2017; UN Environment 2019).

Mercury has long been recognized as an important environmental problem in New York State (Bloomfield et al. 1980). Factors that contribute to this condition include: the location downwind of the many coal-fired power plants in the Ohio River Valley; extensive forested lands particularly in the Adirondacks and Catskills that enhance deposition; an abundance of wetlands that facilitate methylation; and numerous streams, rivers, lakes, and estuaries with extensive fish populations and other wildlife that are exposed to methylmercury (MeHg) through trophic transfer (Driscoll et al. 2007). As a result, there are many Hg-related fish consumption advisories for specific inland freshwaters, the two Great Lakes (Erie, Ontario), and coastal areas of New York State, as well as blanket advisories for the Adirondacks and Catskills (https://www.health.ny.gov/environmental/outdoors/fish/health_advisories/). Despite these challenges, efforts are underway to curb Hg pollution.

Under the Great Lakes Water Quality Agreement, Environment Canada and the U.S. Environmental Protection Agency (US EPA) signed the Great Lakes Binational Toxics Strategy in 1997 calling for virtual elimination of Hg emissions originating from human sources in the Great Lakes region (US EPA 1997). The Great Lakes Regional Collaboration built on this effort and in 2010 produced the Great Lakes Mercury Emission Reduction Strategy with recommendations for decreasing emissions from the largest remaining sources in the basin. The Mercury Air Toxics Standards (MATS) rule went into effect in 2015 with a goal of achieving 91% reductions in Hg emissions from electric utilities (https://www.epa.gov/mats). At the global scale, the Minamata Convention on Mercury entered into force in August 2017, which includes the United States as a ratified Party for this international treaty (Evers et al. 2016).

To advance scientific and public understanding and to inform policy efforts, the New York State Energy Research and Development Authority (NYSERDA) sponsored a scientific synthesis on Hg in atmospheric deposition, water, invertebrates, fish, and wildlife in New York State. The result of this scientific collaboration is a series of 23 papers published in *Ecotoxicology* (Table 1). The findings from these studies are summarized here in the context of an extensive statewide database of Hg concentrations in atmospheric deposition, surface waters, and in a broad array of biota including fish, birds, invertebrates, and others that were compiled as part of the scientific synthesis. The database is presented in summarized form and results from the 23 papers are integrated into this summary article as we answer a series of questions about Hg in New York State including the concentrations that occur and associated risks, spatial and temporal patterns, and policies that are addressing exposure risks.

**Table 1:** Contributed papers for this special issue, their locations with respect to ecoregions in New York State and topical focus

Paper reference #	Lead author	Manuscript title	New York State Ecoregion	Topical focus
1	Adams	The effects of climate, habitat and trophic position on methylmercury availability for breeding New York songbirds	Statewide	Songbirds
2	Buxton	The influence of biotic and abiotic factors on banded common loon (Gavia immer) reproductive success in a remote, mountainous region of the northeastern United States	Northeastern Highlands (Adirondacks)	Aquatic birds
3	Dekenberger	Watershed Influences on Mercury in Tributaries to Lake Ontario	Eastern Great Lakes Lowlands	Water
4	DeSorbo	Bald eagle mercury exposure varies with region and site elevation in New York, USA	Northeastern Highlands (Adirondacks and Catskills)	Aquatic birds
5	Dzielski	Reconstructing avian mercury concentrations through time using museum specimens from New York State	Statewide	Birds
6	Grieb	Assessment of Trends in Fish Tissue Mercury Concentrations	Statewide	Fish
7^a^	Lane	Long-term monitoring of mercury in adult saltmarsh sparrows breeding in Maine, Massachusetts and New York, USA 2000–2017	Atlantic Coastal Pine Barrens	Songbirds
8	Millard (a)	The impact of lime additions on mercury dynamics in stream chemistry and macroinvertebrates: A comparison of management strategies	Northeastern Highlands (Adirondacks)	Invertebrates
9	Millard (b)	Patterns and trends of fish mercury in New York State	Statewide	Fish
10	Nelson	Dragonfly larvae as biosentinels of Hg bioaccumulation in Northeastern and Adirondack lakes: relationships to abiotic factors	Northeastern Highlands (Adirondacks)	Invertebrates
11^a^	Perkins	Historical patterns in mercury exposure for North American songbirds	Statewide	Songbirds
12	Razavi	Mercury concentrations in fish and invertebrates of the Finger Lakes in central New York	Eastern Great Lakes Lowlands (Finger Lakes)	Fish and invertebrates
13	Richter	Mercury in the fish of New York’s Great Lakes: A quarter century of near stability	Eastern Great Lakes Lowlands (Lakes Erie and Lake Ontario)	Fish
14	Riva-Murray (a)	Mercury in Fish from New York’s Streams and Rivers: Spatial Patterns, temporal trends, and environmental driver	Northeastern Highlands (Adirondacks and Catskills)	Fish
15	Riva-Murray (b)	Methylmercury—Total Mercury Ratios in Predator and Primary Consumer Insects from Adirondack Streams (New York, USA)	Northeastern Highlands (Adirondacks)	Invertebrates
16	Sauer (a)	Mercury Exposure in Songbird Communities within Sphagnum Bog and Upland Forest Ecosystems in the Adirondack Park (New York, USA)	Northeastern Highlands (Adirondacks)	Songbirds
17	Sauer (b)	Mercury Exposure in Songbird Communities along an Elevational Gradient on Whiteface Mountain, Adirondack Park (New York, USA)	Northeastern Highlands (Adirondacks)	Songbirds
18	Schoch	Spatial patterns and temporal trends in mercury concentrations in common loons (Gavia immer) from 1998 to 2016 in New York’s Adirondack Park: Has this top predator benefitted from mercury emission controls?	Northeastern Highlands (Adirondacks)	Aquatic birds
19	Swinton	Mercury Increase in Lake Champlain Fish May Be Linked to Extreme Climatic Events	Eastern Great Lakes Lowlands (Lake Champlain)	Fish
20	Taylor	Temporal trends in fish mercury concentrations in an Adirondack Lake managed with a continual predator removal program	Northeastern Highlands (Adirondacks)	Fish
21	Wang	Seasonal Patterns of Total and Methyl Mercury Concentrations in Ground and Surface Waters in Natural and Restored Freshwater Wetlands in Northern New York	Eastern Great Lakes Lowlands (St. Lawrence corridor)	Water
22	Yang	Determining optimal sampling strategies for monitoring mercury and reproductive success in common loons in the Adirondacks of New York	Northeastern Highlands (Adirondacks)	Aquatic birds
23	Ye	A Modeling Study on Impacts of Meteorological Variation and Anthropogenic Emission Reductions on Atmospheric Mercury Input to Upstate New York Ecosystems	Eastern Great Lakes Lowlands (Lake Erie Lake and Ontario)	Atmospheric deposition

^a^These two papers are part of the special paper contributions of this project but are already published in the September 2020 issue of the journal Ecotoxicology

## Study area

**Fig. 1:**
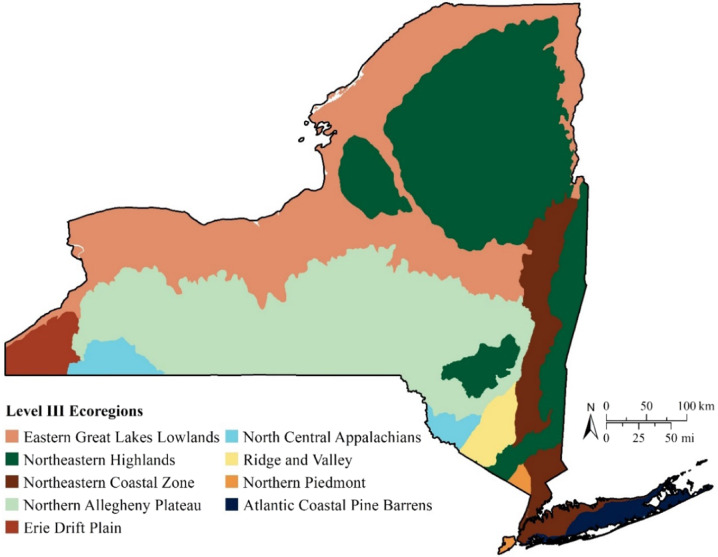
Ecoregions across New York State, United States (Data obtained at: https://www.epa.gov/eco-research/level-iii-and-iv-ecoregions-continental-united-states)

**Fig. 2:**
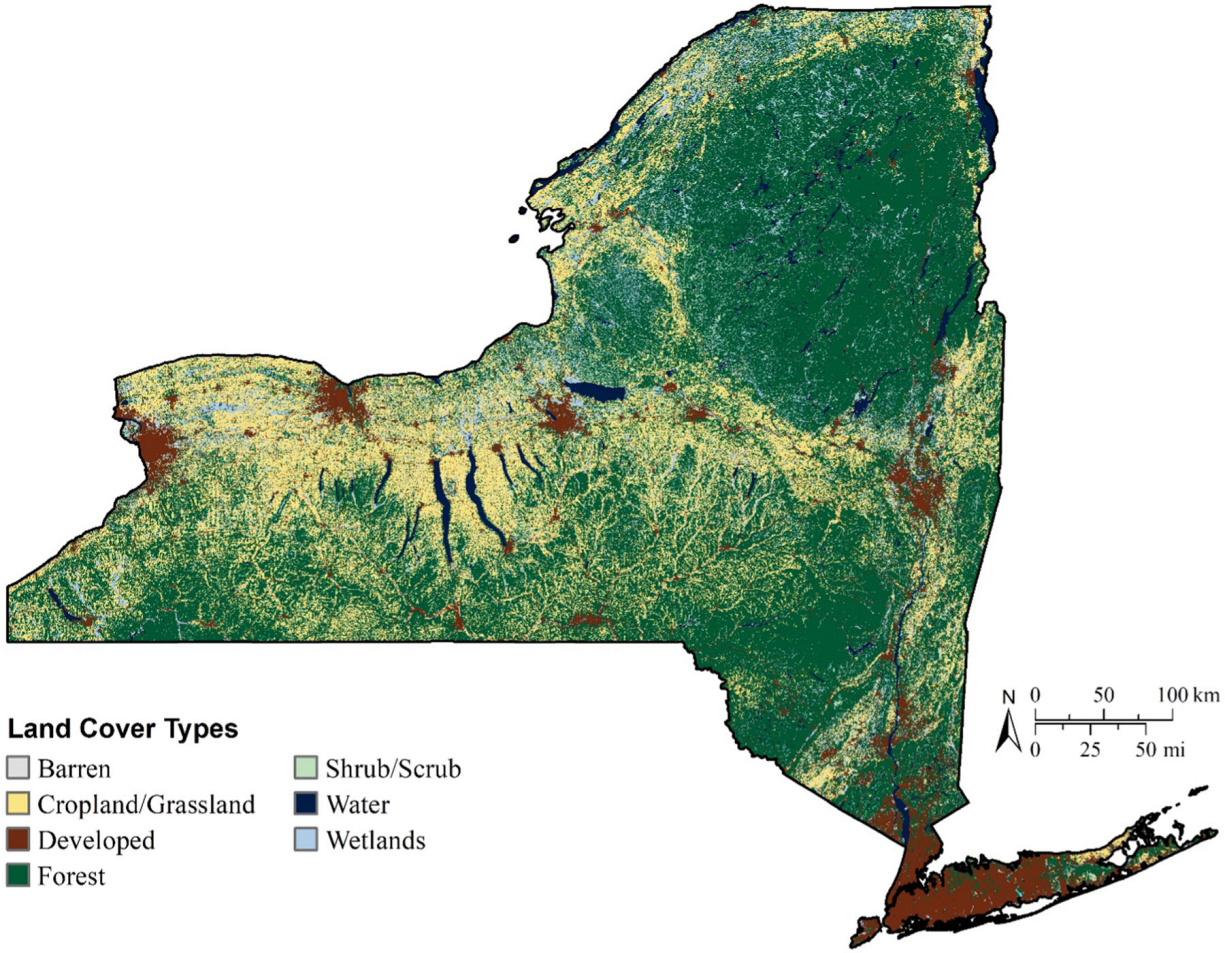
Land cover types of New York State. The state has more than 7600 freshwater lakes, ponds, and reservoirs, as well as portions of Lake Erie and Lake Ontario and over 112,000 km of rivers and streams. After the Great Lakes, New York’s largest lakes include Champlain, Oneida, Seneca, and Cayuga Lakes. Seneca and Cayuga Lakes are part of a series of long, narrow lakes known as the Finger Lakes located in central New York. The State’s largest rivers include the St. Lawrence, Susquehanna, Allegheny, Hudson, and Delaware Rivers (Data obtained at: https://www.usgs.gov/centers/eros/science/national-land-cover-database?qt-science_center_objects=0#qt-science_center_objects)

The USEPA has identified 104 ecoregions across the U.S. In New York, USEPA Level III Ecoregions divide the state into similar lands based on patterns in the mosaic of biotic, abiotic, aquatic, and terrestrial ecosystem components (Fig. 1). These ecoregions provide a spatial framework to evaluate Hg exposure patterns of fish and wildlife that are adapted to these different environments (Fig. 2).

## Methods

### Sampling and measurement units

Observations of Hg concentrations in abiotic media (air, water) and biotic samples (invertebrates, fish, amphibians, reptiles, birds, and mammals) from New York State are available from 1969 through 2017; the total sample size within the New York State Hg database described in this paper was over 70,000 observations of Hg concentrations (including over 47,000 from biota—Table 2). These data comprise the “NYSERDA Synthesis of Environmental Mercury Loads in New York State (1969–2017)” (NYSMD), which is available at Open NY (see Acknowledgments section for URL). Mercury biota data were standardized for each major taxonomic group to a common tissue type (Table 2). For most species, total mercury (THg) was measured and predominantly occurs as MeHg, but in species where MeHg is a smaller fraction of THg, MeHg concentrations were directly measured and are identified within this paper when relevant. Mercury in biotic samples is generally reported in parts per million (ppm) of wet weight (ww).

**Table 2:** Biota sampled in New York State from 1969 to 2017 and standardized tissue type that were used for 20 papers within this special issue reporting biological data

Taxa	Tissue	Hg type/unit	Sample size
Mollusks and other invertebrates	Whole body/muscle	THg, MeHg/ppm, ww	2733
Fish	Whole body/muscle	THg/ppm, ww	33,502
Amphibians	Muscle	THg/ppm, ww	109
Reptiles	Scute	THg/ppm, fw	96
Bird: invertivores	Blood	THg/ppm, ww	8101
Bird: piscivores/carnivores	Blood	THg/ppm, ww	1650
Mammal: invertivores	Fur	THg/ppm, fw	486
Mammal: piscivores	Fur	THg/ppm, fw	511
Total sampled			47,188

The abbreviation *fw* refers to fresh weight without any drying of tissue prior to analysis (usually used for keratin-based tissues), whereas *ww* refers to analyses of tissues with a significant moisture content. Dry weight (*dw*), although not used here, refers to analyses of tissues after extracting all moisture

### Spatial grid system

**Fig. 3:**
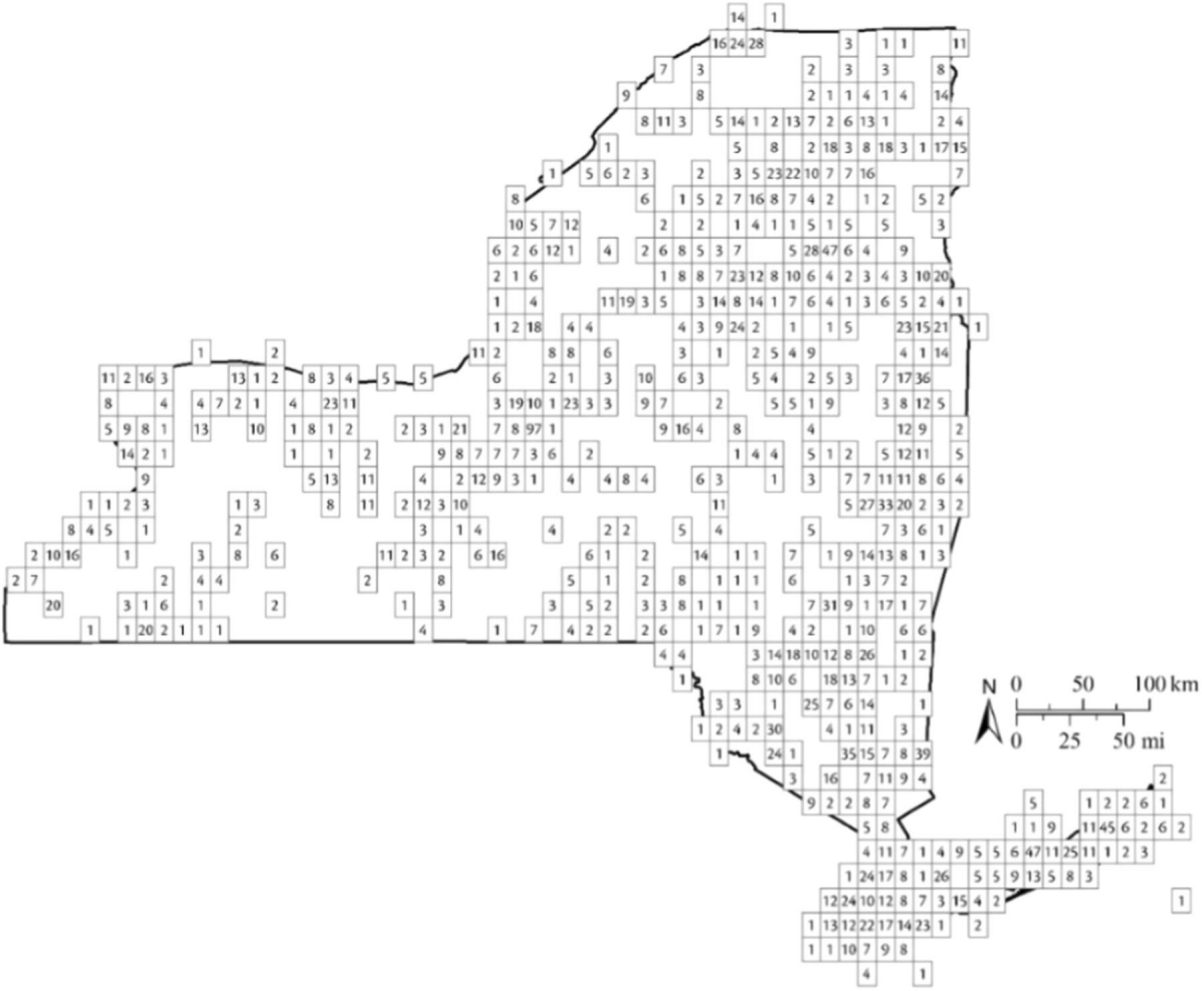
The spatial grid system (1/8 by 1/8 degree grid cells used for defining spatial gradients of biotic Hg concentrations in New York State. The number in each cell represents the number of species of fish and wildlife with Hg observations in the area

To examine Hg patterns in biota (i.e., fish, birds) across New York, the state was divided into 1/8 by 1/8 degree grid cells, each of which represents approximately 250 square kilometers. The number in each grid cell in Fig. 3 depicts the number of wildlife species sampled for Hg. This approach was used in past assessments of effects of Hg loads including biota for the Great Lakes (Evers et al. 2011).

The data from the NYSMD were overlaid across the spatial grid system after standardization of Hg data (Fig. 3). All THg observations were first standardized to a representative tissue type for each taxonomic group. When relevant, bird observations were converted to blood THg (ppm, ww) (Eagles-Smith et al. 2009; Evers et al. 2011). All fish THg data were standardized to whole fish THg (ppm, ww) and were length-standardized to control for variation attributable to size and age using a species-specific general linear mixed model (Buck et al. 2019).

#### Spatial analyses

A spatial analyses was conducted to explore variation of fish Hg concentrations across the state in the context of human and wildlife health risk, with a focus on the landscape characteristics that affect spatial patterns. Two subsets of the NYSMD were created for the spatial analysis. The first included all fish data, while the second subset selected for game fish. These two datasets were then independently analyzed with a general linear mixed model fit in a Bayesian framework using packages *rstan* and *rstanarm* (Carpenter et al. 2017; Goodrich et al. 2018; Stan Development Team 2018) within the R statistical modeling environment (v 3.5.1; R Core Team 2018). Foraging guild and tissue type were fixed effects, while sampling year and species were random effects nested within grid cell as a spatial covariate. The model predicted mean THg concentrations for all fish at an omnivore or higher trophic level for each grid cell where a fish was sampled and were then mapped across New York State. See Adams et al. (2020) for further information on data compilation, standardization, and model development. To better understand the implications of these results for human health, the fish THg concentrations are presented in relation to effect level thresholds published jointly by the US Food and Drug Administration and US EPA (FDA/EPA 2019; see Table 3 for consumption guidelines).

**Table 3:** Fish Hg concentrations and meal frequency guidelines^a^

Guideline or criterion by agency/entity	Mercury in fish (ppm, ww)	Fish consumption guideline
New York State Department of Health	<1.0: general and sensitive population	Four meals per month
	≥1.0–<2.0: sensitive population	No consumption
	≥1.0–<2.0: general population	One meal per month
	>2.0: general and sensitive populations	No consumption
Great Lakes Consortium for Fish Consumption Advisories (for both sensitive and general populations)^b^	0.0–≤0.05	Unrestricted
	0.05–0.10	Two meals per week
	0.11–0.21	One meal per week
	0.22–0.95	One meal per month
	>0.95	No consumption
USEPA—Food and Drug Administration Fish Advice: technical information^c^	≤0.15	Three meals per week
	0.16–0.22	Two meals per week
	0.23–0.46	One meal per week
	>0.46	No consumption

^a^Mercury concentrations are interpreted in the context of the number of fish meals that could be consumed to stay within the USEPA health-based reference dose for MeHg (USEPA 2001)

^b^GLRC 2010

^c^USEPA; https://www.epa.gov/fish-tech/epa-fda-fish-advice-technical-information#

Additionally, landscape scale variables play an important role in Hg availability to biota. Mercury concentrations in fish are driven in part by the Hg inputs (e.g., atmospheric deposition), methylation rates, and trophic level. A portion of the atmospherically deposited Hg is converted to its bioavailable form, MeHg. Landscape characteristics can amplify this conversion process to contribute to the spatial heterogeneity of Hg across the landscape. A spatial analysis of Land Use and Land Cover (LULC) data for New York State was conducted to highlight these relationships. The percent cover of forest and agriculture were extracted, for each grid cell, from the National Land Cover Database (NLCD) and, where grid cells overlapped spatially with Canada, the Land Cover of Canada database (Latifovic et al. 2017; Yang et al. 2018). A simple linear model was then constructed to examine the relationship between game fish Hg and LULC (forest and agriculture, respectively) by grid cell.

#### Temporal analyses

Temporal analyses were conducted to assess changes in Hg concentrations for 15 well-sampled (>10 total years of sampling, >400 total samples) indicator species (11 fish and four birds) by USEPA Level III Ecoregions in New York State. This dataset was also analyzed using a general linear mixed model with the same framework as the spatial analysis. The temporal model used tissue type as a fixed effect, while the nested effect of ecoregion, species, and year were random effects. The output of this model predicted a species-specific mean Hg concentration annually for each ecoregion, year combination sampled for that respective species (while controlling for grid-cell level spatial variation). These predicted values were then input into a series of linear models to examine species-specific trends in Hg concentrations through time for each ecoregion, species combination with more than 2 years of sampling. See Adams et al. (2020) for further information on data compilation, standardization, and model development.

## Results and discussion

Measurements of environmental Hg concentrations and fluxes improve understanding of Hg dynamics and effects in the environment and help parameterize models that simulate and project fate and exposure of Hg. Through a collaborative effort, this special issue of *Ecotoxicology* includes 23 papers representing a wide variety of Hg studies across New York State including atmospheric deposition (one paper), surface waters (two papers), invertebrates (three papers), fish (seven papers) and birds (ten papers) (Table 1). These papers collectively characterize environmental Hg deposition and concentrations in ecosystems of New York State, evaluate patterns of methylation, determine bioaccumulation and exposure in aquatic and terrestrial food webs, and address temporal trends of Hg in the context of changes in emissions and atmospheric deposition using various biota. This work also places Hg contamination in New York State in context with other areas of the U.S (Grieb et al. 2020; Olson et al. 2020). Integrating the findings from these papers provides a robust assessment of the impact of Hg on ecosystems of New York State, as well as a broader scientific basis for policymakers and natural resource managers to make informed decisions regarding future air regulations and fish consumption advisories.

Our findings about the extent and effects of Hg pollution in New York State are described in the context of three policy-relevant questions: (1) What are the sources of Hg inputs to New York State and how do these inputs vary over space and time?; (2) What risks does mercury pollution pose to humans in New York State?; and (3) What are the spatial and temporal patterns of Hg in biota in New York State and to what extent do these reflect sensitivity to Hg inputs and changes in those inputs? Specific science-policy connections are addressed at the end of this paper.

### What are the sources of mercury inputs to New York State and how do these inputs vary over space and time?

#### Mercury emission sources that affect New York State

Initial regulatory efforts in the 1970s focused on large industrial sources of Hg, such as chlor-alkali plants. These point sources discharged Hg directly or indirectly into the Great Lakes and their tributaries. Today, many of these sources have been reduced or eliminated, which has led to a partial recovery from point-source Hg pollution (Evers and Clair 2005; Cain et al. 2011; Drevnick et al. 2012; Todorova et al. 2014). Atmospheric emissions and deposition are currently the largest source of Hg to New York State. Stationary sources (e.g., fossil fuel burning plants and waste incinerators) emit Hg into the atmosphere as gaseous elemental Hg, gaseous oxidized Hg, or particulate bound Hg. Gaseous elemental Hg can be transported long distances (atmospheric residence time ~0.5–1 yr), while gaseous oxidized Hg and particulate bound Hg tend to be deposited near the source (hours to days). For this reason, Hg deposition to New York State can originate from local, regional, national, or global sources.

**Fig. 4:**
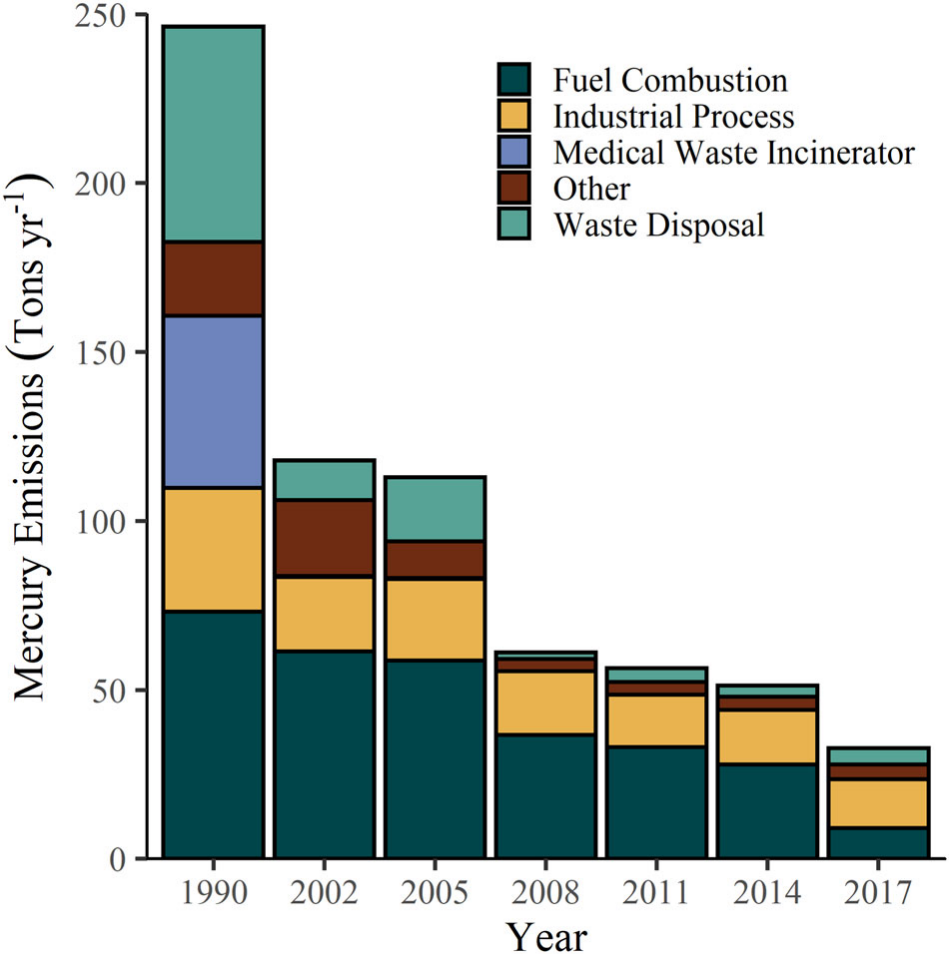
US EPA National Emissions Inventory (NEI) estimates of Hg emitted to the air by major sources in the United States from 1990 to 2017. Coal-fired power plants remain the largest source of Hg emissions in the US (USEPA 2020)

**Fig. 5:**
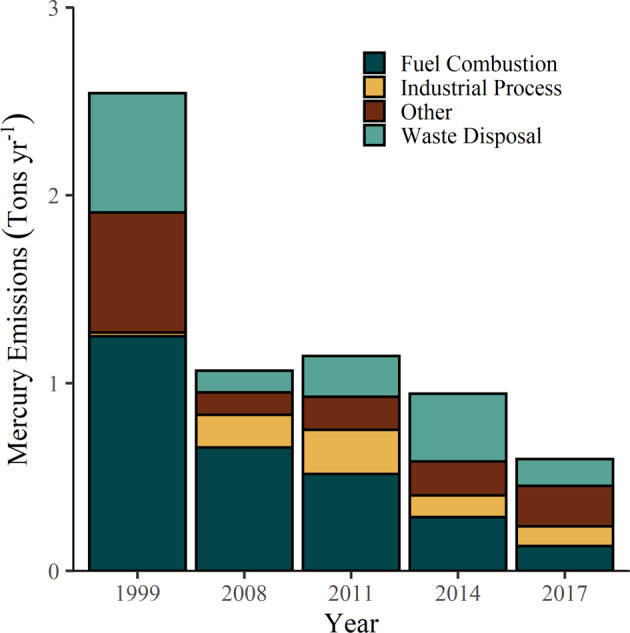
US EPA NEI estimates of Hg emitted to the air by major source in New York State from 1999 to 2017 (USEPA 2020)

Controls on Hg emissions in the US were first initiated by designation of Hg as a hazardous air pollutant through the 1990 Amendments of the Clean Air Act (CAA). The US EPA was required to list and set emissions standards which later resulted in a finding that it was appropriate and necessary to regulate Hg emissions from coal- and oil-fired power plants and other sources such as incinerators (Milford and Pienciak 2009). This began a regulatory process which has led to a more than four-fold decline in U.S. anthropogenic Hg emissions between 1990 and 2017 (Olson et al. 2020). During 1990 to 2005, the largest decreases in Hg emissions were from hospital and municipal incinerators (96–99% decrease) and chlor-alkali facilities (97% decrease; Schmeltz et al. 2011). Mercury emissions from electric utilities have declined since the 1990s due to a variety of state and federal regulations, many of which affected emissions indirectly as co-benefits of controlling other pollutants such as sulfur dioxide and nitrogen oxides (Milford and Pienciak 2009; Schmeltz et al. 2011; Zhang et al. 2016) and in recent years following the implementation of the MATS rule. The average rate of decline of Hg emissions from electric utilities was −7.4 percent per year during 1996 to 2017 (Olson et al. 2020). In the U.S., approximately 52 tons of Hg were annually emitted from anthropogenic sources in 2014 (Bourtsalas and Themelis 2019), decreasing to 33 tons in 2017. Emissions of Hg from coal-fired power plants have decreased to the point that they are no longer the largest source of anthropogenic atmospheric Hg emissions in the U.S., being replaced by industrial processes (Fig. 4). There are major and minor sources of anthropogenic Hg emission sources across New York State, the largest Hg emission source is “other categories” which include nonpoint emissions and point source emissions associated with various industries (Fig. 5). In contrast to declines in New York State and the U.S., inventories suggest that global anthropogenic Hg emissions have increased in recent years (Streets et al. 2019; UN Environment 2019). Asia continues to be a dominant contributor to Hg emissions globally, due largely to expanding energy production from coal-fired power plants and gold mining.

Source attribution of Hg has been conducted for New York State and the Northeast. Lin et al. (2012) conducted source apportionment of Hg emissions for the contiguous U.S. using the Community Multiscale Air Quality (CMAQ) model. In New York and New England, relatively high contributions of Hg were derived from electrical generating units and waste incineration. Sources outside the U.S. also contributed to Hg deposition in New York and New England but the fractions were smaller than in many other regions of the U.S. Choi et al. (2008) conducted source contribution analysis for forms of atmospheric Hg in the Adirondacks observing that Pennsylvania, West Virginia, Ohio, Kentucky, Texas, Indiana, and Missouri were important contributing areas.

#### Spatial and temporal patterns in atmospheric Hg concentrations and deposition

**Fig. 6:**
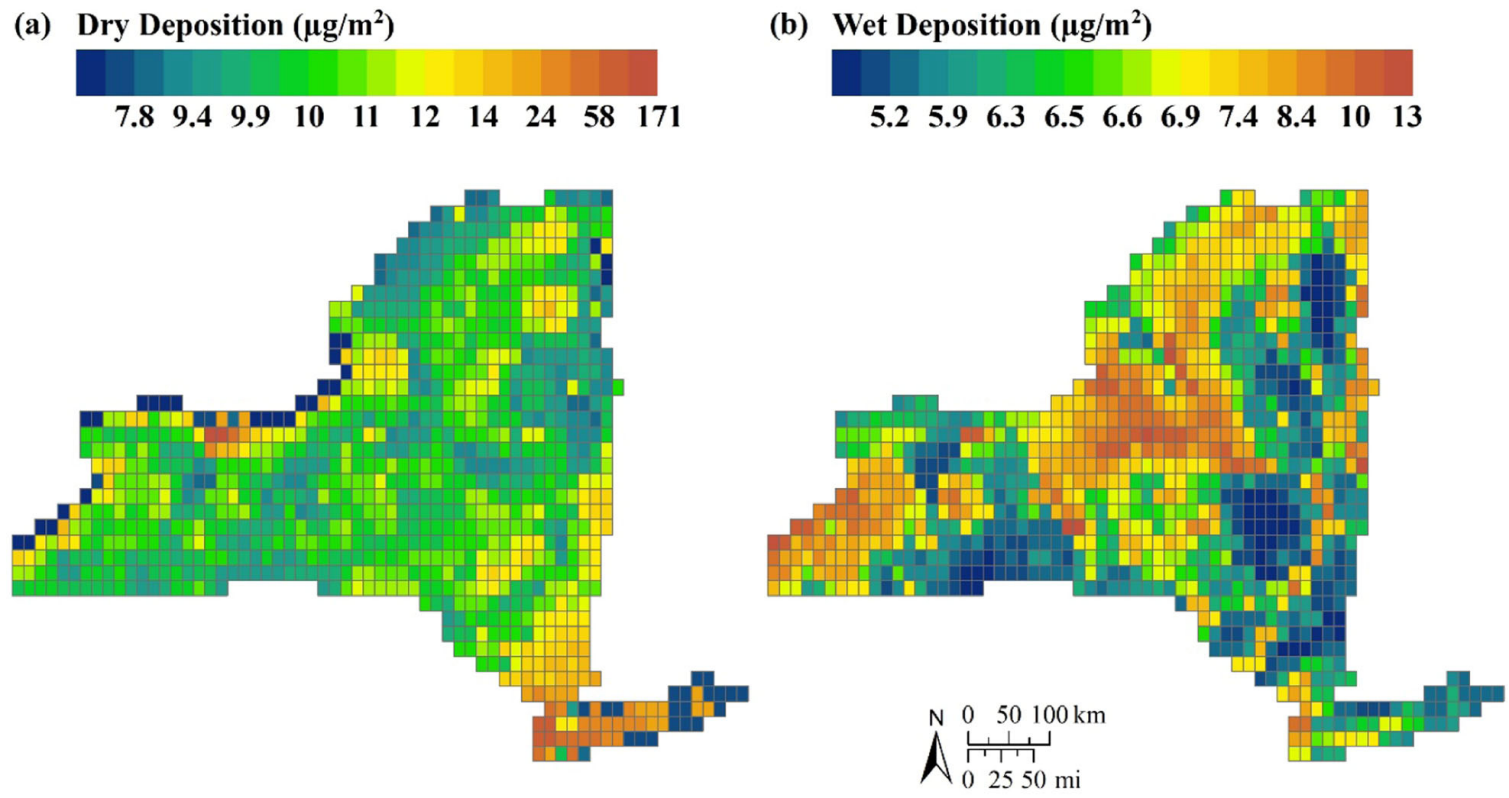
Simulated spatial patterns of dry and wet Hg deposition across New York State based on Ye et al. (2020) at a 1/8 by 1/8 degree grid scale. Patterns of (**a**) dry and (**b**) wet Hg deposition vary considerably across the state due to emissions point sources, precipitation, wind speed, heat flux, and land cover. The z-axes for dry and wet deposition are shown on different scales

Ye et al. (2020) modeled Hg deposition across New York State, using CMAQ with state-of-the-science air chemistry algorithms. They examined the contribution of local emissions and the role of environmental variables to spatial patterns of Hg deposition (Fig. 6). Meteorological conditions and forest canopy characteristics were the most important factors affecting spatial patterns in Hg wet and dry deposition, respectively.

*Dry deposition*: Local, anthropogenic Hg emissions sources increase deposition rates over water and land within 50 km of the point source. For example, emission sources near Rochester, in Manhattan, and on Long Island contribute to elevated dry Hg deposition in those areas (Fig. 6a). Environmental factors such as wind speed and heat flux (changes in temperature across the surface of water and land) drive spatial patterns of deposition in aquatic habitats, while increased canopy cover associated with forested lands lead to greater deposition in terrestrial landscapes.

*Wet deposition:* The amount of precipitation is an important driver of spatial variation in wet Hg deposition to aquatic and terrestrial habitats. Higher levels of precipitation downwind of Lake Erie and Lake Ontario lead to increased Hg deposition in the central and western regions of the state (Fig. 6b).

**Fig. 7:**
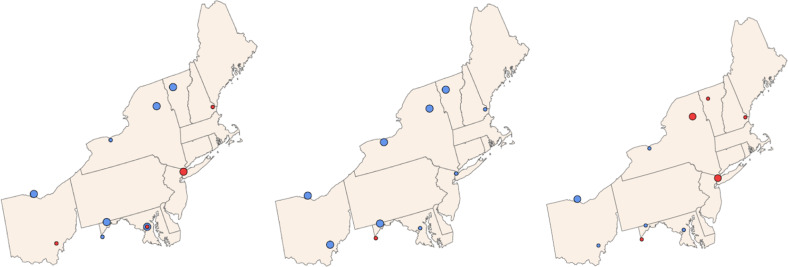
Long-term temporal trends in concentrations of (**a**) gaseous elemental Hg, (**b**) gaseous oxidized Hg, and (**c**) particulate bound Hg for the northeastern U.S. A blue symbol indicates a decreasing trend and a red symbol shows an increasing trend. Large symbols are indicative of a significant trend. Modified after Olson et al. (2020)

In the U.S., concentrations of atmospheric Hg species are monitored by the Atmospheric Mercury Network (AMNet; http://nadp.slh.wisc.edu/amnet/). In New York State, there are three AMNet sites of the National Atmospheric Deposition Program. Olson et al. (2020) examined temporal trends in atmospheric Hg species generally finding decreases in concentrations of gaseous elemental Hg (7a) and gaseous oxidized Hg (7b) in the Northeast from 2008 to 2017, which are consistent with regional and national decreases in emissions. Trends for particulate bound Hg are more varied with some sites in New York State showing increases in concentrations (7c), which may be caused by wood burning in winter (Zhou et al. 2017) (Fig. 7).

**Fig. 8:**
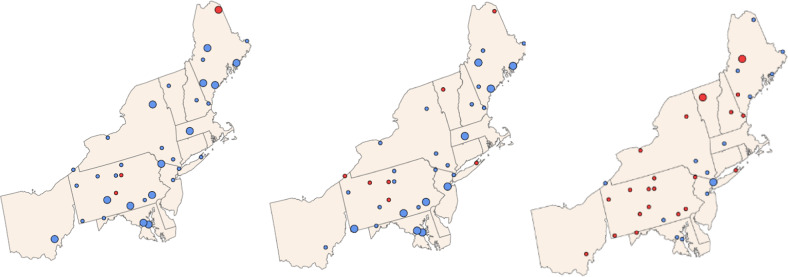
Long-term trends in (**a**) annual volume-weighted concentrations of Hg in wet deposition, (**b**) annual wet Hg deposition and (**c**) annual precipitation quantity for the northeastern U.S. from the Mercury Deposition Network. A blue symbol indicates a decreasing trend and a red symbol shows an increasing trend. Large symbols are indicative of a significant trend. Modified after Olson et al. (2020)

Trends in wet Hg deposition are monitored by the Mercury Deposition Network (MDN; http://nadp.slh.wisc.edu/mdn), of the National Atmospheric Deposition Program, including six sites in New York State. Olson et al. (2020) evaluated long-term temporal trends in wet Hg deposition during 1996 to 2017. They found generally consistent decreasing trends in annual volume-weighted concentrations for stations in the eastern U.S., including all of those in New York State (Fig. 8a) which is generally consistent with decreases in U.S. emissions. Trends in annual wet Hg deposition are less than those of concentrations, although many sites in the Northeast show significant decreasing trends (Fig. 8b). This diminished trend is due to considerable year-to-year variability in precipitation quantity and a general pattern of increasing precipitation quantity in the Northeast (Fig. 8c).

Landscape factors have been shown to exert important controls on Hg deposition. In particular, the forest canopy mediates land-atmosphere exchange of Hg. Elemental Hg enters the stomata of leaves and is later deposited to soil as litterfall. Ionic Hg can be adsorbed to the surface of leaves and needles and washed off during subsequent rain events and deposited as throughfall. In New York State, studies have shown that in hardwood stands litterfall is the largest pathway of Hg deposition to the forest floor (Blackwell et al. 2014). However, Hg deposition is typically greater in coniferous stands than in hardwoods because of the persistence of needles throughout the year and the high leaf area index of the coniferous canopy, and throughfall is the dominant pathway of Hg deposition (Blackwell et al. 2014). Finally, Hg deposition increases with elevation due to increases in precipitation quantity and shifts in vegetation (Blackwell and Driscoll 2015). At high elevations, cloud deposition can dominate atmospheric Hg input (Gerson et al. 2017). Soil and surface water Hg concentrations increase with elevation, likely reflecting increases in deposition (Blackwell and Driscoll 2015; Millard et al. 2020b).

### What risks to humans does mercury pollution pose in New York State?

Fish are well-established bioindicators of environmental Hg loads and conditions that facilitate the transport, transformations and trophic transfer of Hg. Fish data are used to understand long-term temporal trends in Hg (Millard et al. 2020b; Riva-Murray et al. 2020a; Richter and Skinner 2020; Swinton and Nierzwicki-Bauer 2020), evaluate human health concerns (Grieb et al. 2020), link to land management and water quality (Millard et al. 2020b; Razavi et al. 2020), and inform fisheries management (Taylor et al. 2020). Fish Hg data are also used to inform policy and management in New York State.

#### Mercury exposure—risks to humans

Human populations most at risk of MeHg exposure include: (1) sensitive individuals (e.g., women of childbearing age, pregnant women, and children); and (2) people whose diets include large amounts of high trophic-level fish (e.g., recreational anglers and subsistence fish consumers). The greatest risks to humans from dietary uptake of MeHg are observed with high consumption levels of upper trophic level species. For example, primary consumers (e.g., shellfish such as mussels) at trophic level 2 have relatively low MeHg concentrations and are considered safe for consumption. Secondary consumers (e.g., salmon, herring) are a trophic step higher, but are also healthy choices.

For tertiary or higher consumers, which include predatory fish (e.g., bass, walleye, bluefish), MeHg concentrations can be elevated to levels that raise human health concerns. The variability of concentrations in trophic level 4 fish can be related to size and species. Therefore, large trophic level 4 fish are the best bioindicators to assess potential exposure risk of Hg to humans. In coastal waters of New York State and nearby regions, large marine predatory fish such as tuna, swordfish and shark can have elevated MeHg concentrations, frequently exceeding the no consumption limits identified by the USEPA (i.e., 0.46 ppm, ww) and the Great Lakes Consortium (i.e., 0.95 ppm, ww) (Karimi et al. 2012, 2013; Lee et al. 2016).

New York State fish consumption advisories are issued by the New York State Department of Health (DOH) and are based on a risk management approach and guidelines (Table 3). If there is no specific advice for a water body, a general statewide advisory that recommends limiting sport fish consumption to up to four meals per month applies (because fish from all waters have not been tested, and fish may contain contaminants other than those that are routinely tested). In most cases, if a water body has a specific consumption advisory for the general population (men and older women), the sensitive population (women under 50 and children under 15) are advised not to consume any fish from that water body. Women under 50 and children under 15 are also advised to avoid consuming predator fish species from all water bodies in the high Hg regions of the Catskills and Adirondacks. There are fish consumption advisories for Hg in 92 water bodies New York State, with 62 of these in the Adirondacks (https://www.health.ny.gov/environmental/outdoors/fish/health_advisories/).

#### Mercury Concentrations in waters of New York State

**Fig. 9:**
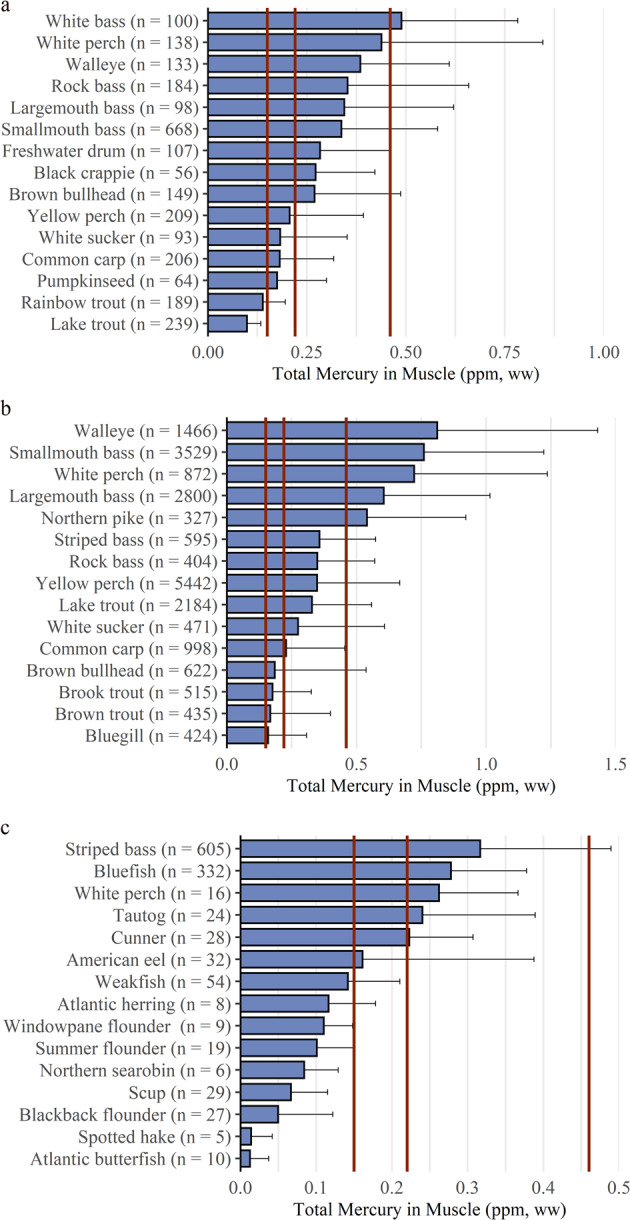
**a** Mean concentrations and standard deviation of Hg in fish of Lake Erie and Lake Ontario of New York State (*n* = 2633). Three vertical lines indicate consumption advisory levels. The 0.15 and 0.46 ppm vertical lines bracket the USEPA advisory levels. The mean Hg concentration of 9 of 15 species exceed the 0.22 ppm health threshold suggested by the Great Lakes Consortium (Table 3). **b** Mean concentrations and standard deviation of Hg in fish of inland waters of New York State (*n* = 21,084). Three vertical lines indicate consumption advisory levels. The 0.15 and 0.46 ppm vertical lines bracket the USEPA advisory levels. The mean Hg concentration of 11 of 15 species exceed the 0.22 ppm health threshold of the Great Lakes Consortium (Table 3). **c** Mean concentrations and standard deviation of Hg in fish of nearshore marine waters of New York State (*n* = 1204). Three vertical lines indicate consumption advisory levels. The 0.15 and 0.46 ppm vertical lines bracket the USEPA advisory levels. The mean Hg concentration of 5 of 15 species exceed the 0.22 ppm health threshold of the Great Lakes Consortium (Table 3)

Mercury concentrations in the 15 species of fish with the highest levels in three major aquatic ecosystems of New York State vary by species (Fig. 9). Fish Hg concentrations for Lake Erie and Lake Ontario over the past three decades, remain on average above the 0.22 ppm (ww) advisory threshold suggested by the Great Lakes Consortium for 9 of the top 15 species analyzed (60%), based on the NYSMD (*n* = 2633; Fig. 9a). Fish in the lower Great Lakes with the highest concentrations of Hg include white bass (*Morone chrysops*), white perch (*Morone americana*), and walleye (*Sander vitreus*).

There is considerable variability in fish Hg concentrations in inland waters of New York State, depending on region, species, and type of water body. Understanding this variability is important for interpreting patterns of exposure and monitoring trends for human and ecological health. Fish Hg concentrations in inland waters over the past three decades, remain on average above the 0.22 ppm (ww) advisory threshold for 11 of the top 15 species (73%) based on the NYSMD (*n* = 21,084; Fig. 9b). Inland water fish with the highest concentrations of Hg include walleye, smallmouth bass (*Micropterus dolomieu*), and white perch. In the Finger Lakes region, fish Hg concentrations of most concern for human consumption are found in walleye and largemouth bass (*Micropterus salmoides*) but were not correlated with aquatic invertebrate MeHg concentrations (Razavi et al. 2020). In a survey of patterns of fish Hg across New York State, Millard et al. (2020b) found the highest concentrations in the Adirondack and Catskill regions.

In coastal marine waters, where most of the human fish consumption diet originates (over 90% in North America; Sunderland et al. 2018), fish Hg concentrations exceed 0.22 ppm (ww) for 5 of the top 15 species analyzed (33%), based on the NYSMD (*n* = 1204; Fig. 9c). Patterns of coastal marine fish Hg are challenging to understand as sampling efforts are less robust than inland waters (Grieb et al. 2020). Chen et al. (2020) investigated the role of nutrient supply in fish Hg concentrations in coastal Long Island. In shallow coastal lagoons of Great South Bay and Jamaica Bay, they found that Hg inputs cycle through sediments which are continuously resuspended into the water column and redeposited back to sediments. They found that nutrient supply alters the uptake of MeHg at the algal base of the food chain through biodilution, while nitrogen appears to subsequently enhance trophic transfer of MeHg to prey fish.

### What are the spatial and temporal patterns of mercury in biota in New York State and to what extent do these reflect sensitivity to mercury inputs and changes in those inputs?

#### Ecosystem sensitivity to mercury

Inorganic Hg enters ecosystems through atmospheric deposition, water releases (e.g., from wastewater treatment facilities, Glass et al. 1990), and leaching from land (e.g., from landfills and industrially contaminated sites; Kocman et al. 2017; Streets et al. 2017; Hsu-Kim et al. 2018; Obrist et al. 2018). Once in the environment, inorganic Hg can be transported to reducing zones (wetlands, sediments) and converted to MeHg by bacteria and archea (Gilmour et al. 2013a, 2013b; Podar et al. 2015). Methylmercury is toxic and can bioaccumulate at the base of the food web and biomagnify through the food chain resulting in elevated concentrations in the tissues of fish, wildlife, and humans, causing adverse health effects.

New York State and much of the Great Lakes basin is a net sink for Hg inputs (Denkenberger et al. 2012), with more Hg entering the basin through emissions and deposition than leaving through re-emission to the atmosphere or drainage losses. As a result, Hg deposited to the region accumulates in soils, and some of this legacy Hg is gradually leached to surface waters. Mercury recently deposited to the landscape tends to be more bioavailable than Hg long buried in soils and sediments (Hintelmann et al. 2002); although, Hg in soils can be mobilized by disturbances, such as storm runoff events, fires, and logging (Willacker et al. 2019; Denkenberger et al. 2020).

**Fig. 10:**
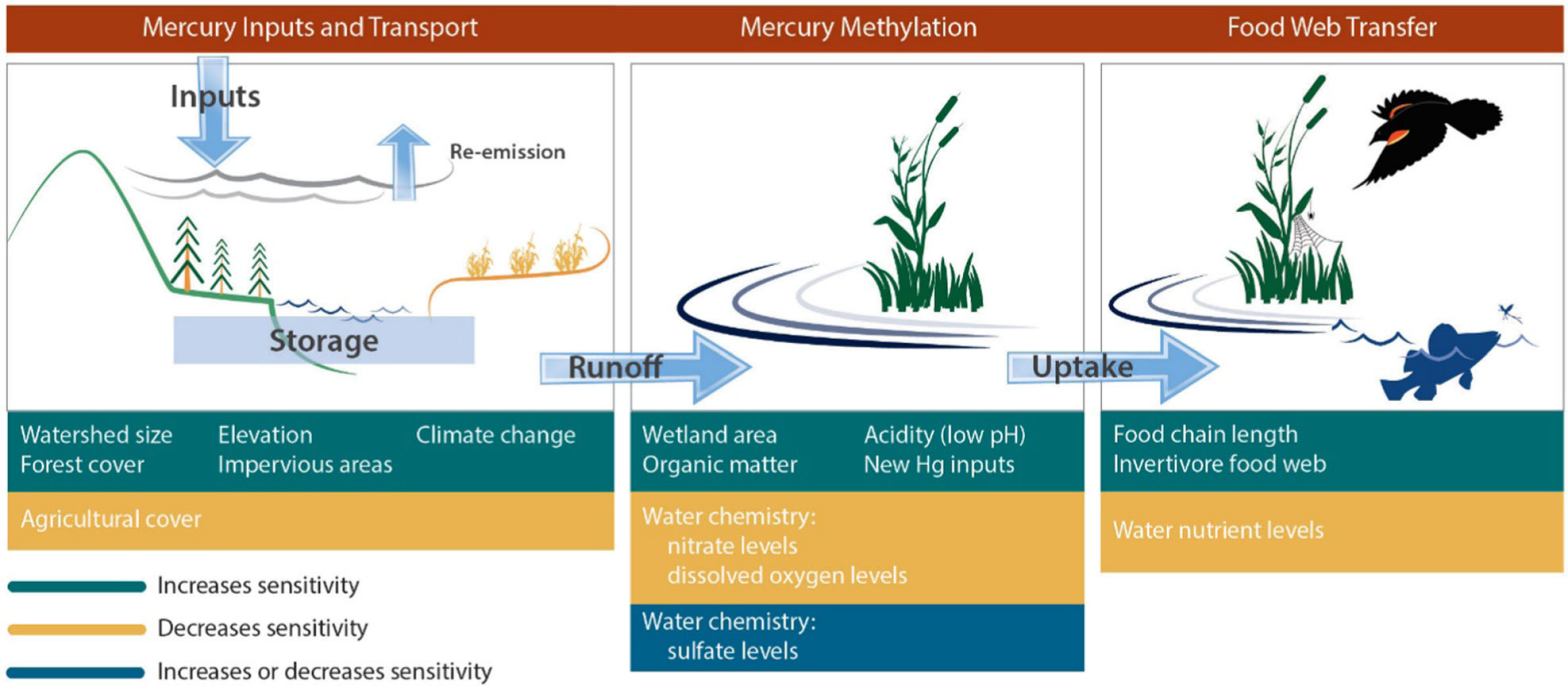
Watershed Hg sensitivity connects landscape features related to Hg input and transport, methylation, and food web transfer for important factors in New York (Driscoll et al. 2007; Yu et al. 2011)

Mercury-sensitive areas with abundant forests receive elevated Hg inputs via throughfall and litterfall from atmospheric emissions and deposition to the forest canopy (Fig. 10). A fraction of the Hg deposited to sensitive landscapes is converted to MeHg largely in wetlands, sediments, and other favorable reducing environments. This conversion process is amplified under conditions of high organic matter, low oxygen, and low pH, that are common in northern forest landscapes (Hsu-Kim et al. 2013; Fig. 10). Sulfate has also been shown to influence MeHg formation. Methylation rates increase up to concentrations of about 20 mg/L and decline under elevated sulfate concentrations in sulfur addition experiments (Gilmour and Henry 1991). However, in natural wetland settings where atmospheric deposition is the primary source of sulfate to the landscape, sulfate availability and MeHg are strongly correlated with no limitation evident (Åkerblom et al. 2013). Conditions where low oxygen, high organic matter, and sulfate reduction occur such as in wetlands, are particularly effective at mobilizing MeHg, which may be further promoted by drying-rewetting cycles (Millard et al. 2020a; Wang et al. 2020). Furthermore, areas that supply elevated dissolved organic carbon (DOC) from decaying organic matter may facilitate the transport of inorganic and MeHg to downstream environments (Dittman et al. 2010; Burns et al. 2012; Millard et al. 2020a) and may be correlated with MeHg concentrations in macroinvertebrates (e.g., odonates; Nelson et al. 2020) and fish (Driscoll et al. 2007). Regions that have been acidified by acid deposition may be particularly sensitive to atmospheric Hg deposition, as sulfate from atmospheric deposition can serve as an important substrate for methylation (Wyn et al. 2009; Coleman Wasik et al. 2015), and Hg strongly bioaccumulates in zooplankton, fish, and loons at low pH (Driscoll et al. 2007; Yu et al. 2011). Finally, the nutrient supply and productivity of the water bodies can influence the concentration of Hg through biodilution and growth dilution (Driscoll et al. 2007, 2012; Chen et al. 2020).

In addition to variation in watershed sensitivity throughout the region, climatic variation and events can affect spatial patterns of Hg concentrations in biota. Swinton et al. (2020) described significant increases of Hg in smallmouth bass and yellow perch (*Perca flavescens*) on Lake Champlain and attributed to increased tributary loading of total suspended solids that resulted from a past storm runoff event.

In areas where atmospheric Hg deposition is moderate, effects on biota may be disproportionately high if conditions exist that promote Hg transport, methylation, and trophic transfer. Conversely, ecosystems with limited ability to transport inorganic Hg to zones of methylation or low methylation potential may have low levels of MeHg in biota despite considerable anthropogenic Hg contamination. The decoupling of inorganic Hg sources with MeHg production and bioaccumulation and biomagnification is evident at local (Evers et al. 2007) and landscape levels (Eagles-Smith et al. 2016). Millard et al. (2020a) found that dissolved organic matter and associated Hg was mobilized from soil to streamwater by watershed liming (calcium carbonate application). However, this enhanced transport did not result in increased MeHg concentrations in streamwater and stream macroinvertebrates due to limited zones of methylation (riparian areas, wetlands) within the watershed. The complexity of the Hg cycle challenges our ability to effectively predict concentrations in upper trophic level fish and wildlife from Hg concentrations in environmental media (soil, water, sediment; Gustin et al. 2016; Sunderland et al. 2016). Identifying appropriate bioindicators based on their relationship with sensitive ecosystems is a critical first step in assessing risk to ecological and human health through long-term Hg monitoring.

#### Fish species

Fish size class reflects different aspects of trophic transfer of Hg: (1) young fish (<1 year) reflect rapid changes in the availability of MeHg and local conditions; (2) mid-sized fish are important for assessing impacts to fish-eating wildlife such as common loons (*Gavia immer*), bald eagles (*Haliaeettus leucocephalus*), osprey (*Pandion haliaetus*), and North American river otter (*Lontra canadensis*); while (3) large fish that are at high trophic levels (i.e., game fish) are of particular concern for human health (Driscoll et al. 1994; Millard et al. 2020b).

The impacts on fish health and reproductive welfare are not well established, but have been summarized previously (Depew et al. 2012a, 2012b; Table 4). While fish Hg concentrations are commonly examined for their impacts on humans (i.e., muscle tissue) or for wildlife exposure (i.e., whole body), the MeHg concentrations in fish tissues also impact behavior, reproductive abilities, and overall health of fish. Fish may exhibit impaired reproductive success at relatively low MeHg concentrations as low as 0.04 ppm (ww) (Depew et al. 2012b) and may have adverse visible behavioral impacts at dietary MeHg concentrations of 0.50 ppm, ww or higher (Depew et al. 2012b). Lower reproductive success reduces the size and sustainability of healthy fish populations, which can have adverse impacts on associated populations of piscivores and human recreational and commercial interests. Unlike freshwater fish, there have been few rigorous published studies evaluating toxicity of MeHg to marine fish (Scheuhammer et al. 2015).

**Table 4:** Screening benchmarks for piscivorous fish and birds for reproductive endpoints

Taxa	Screening benchmark: dietary MeHg (ppm, ww)	Endpoint of interest
Fish	>0.04	Impaired reproductive success (Depew et al. 2012b)
	>0.50	Adverse effects behavior (Depew et al. 2012b)
Birds	0.10–0.18	Adverse effects on behavior (Depew et al. 2012a)
	0.18–0.40	Impaired reproductive success (Depew et al. 2012a)
	>0.40	Reproductive failure (Depew et al. 2012a)

**Fig. 11:**
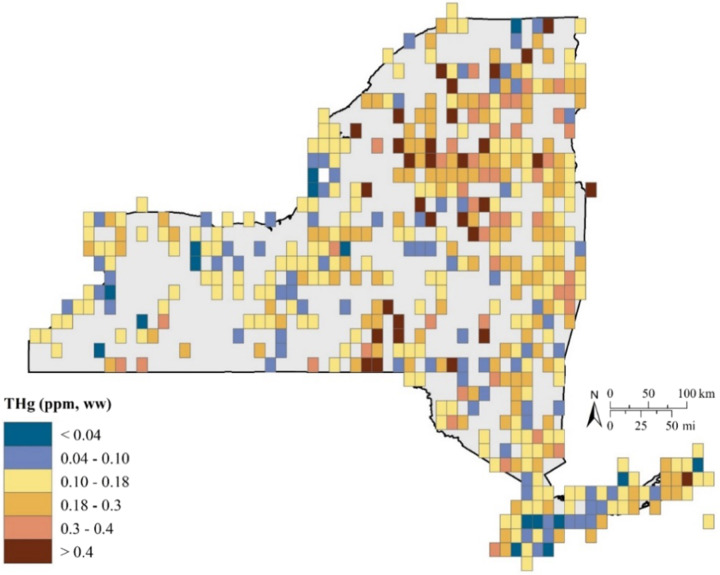
A total of 33,502 fish samples were analyzed for Hg in New York from 1969 to 2017 representing 485 grids for the state (47% represented). Of those grids, 80%, 42%, and 6% had average Hg concentrations above 0.10, above 0.18 and above 0.40, respectively, which are important for the health and reproductive welfare of avian piscivores (Depew et al. 2012a)

Game fish are predatory fish that tend to be at high trophic levels (i.e., trophic levels 4 or 5) and therefore experience several levels of biomagnification of MeHg. Human consumption of game fish in New York State usually includes species such as: walleye from Lake Erie and Lake Ontario; smallmouth and largemouth bass from inland lakes and rivers; and bluefish (*Pomatomus saltatrix*) and striped bass (*Morone saxatilis*) from nearshore marine areas, all of which are well reflected in the NYSMD (Fig. 11).

**Fig. 12:**
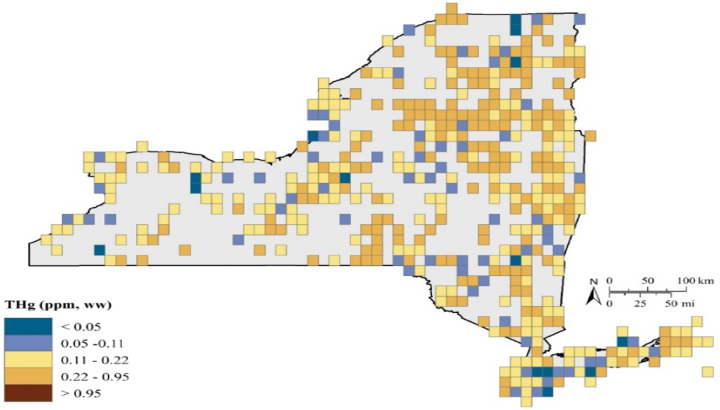
A total of 25,909 game fish samples (38 species) were analyzed for Hg in New York from 1969 to 2017, representing 452 grid cells with Hg exposure data. Of these grids, 43 percent have average game fish Hg concentrations over 0.22 ppm, with the Adirondack and Catskill Mountain regions and upper Susquehanna River Valley regularly having grids with elevated game fish Hg concentrations

**Fig. 13:**
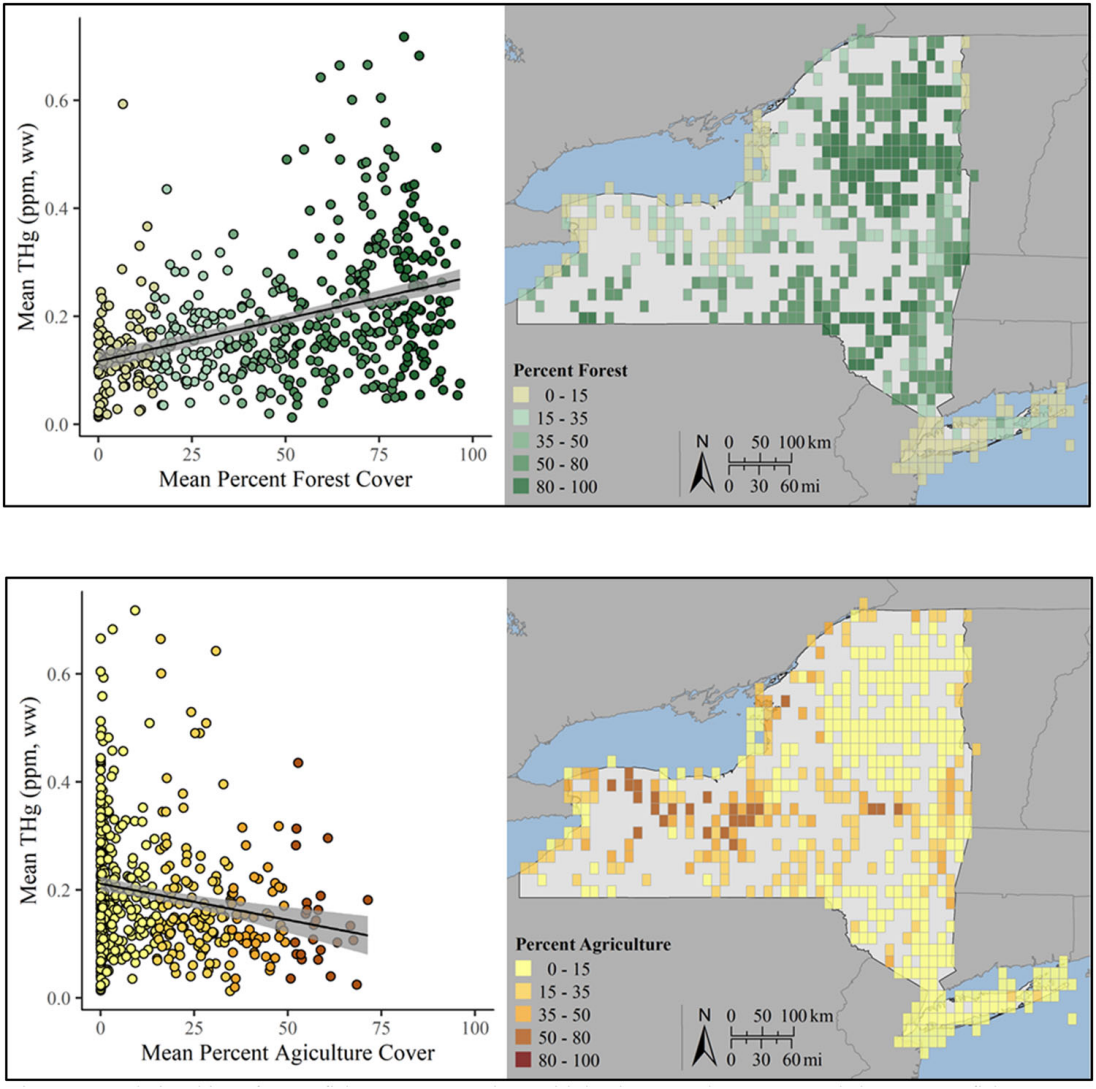
Relationships of game fish Hg concentrations with land cover. The upper panel shows game fish Hg concentrations as a function of forest land cover (Mean Game Fish THg) = 0.117119 + 0.001568 (Mean Percent Forest Cover), *F*_1,483_ = 87.54, *R*^2^ = 0.1517, *p* < 0.001) with spatial distribution forest land cover on the right. The lower panel shows game fish Hg concentrations as a function of agricultural land cover (Mean Game Fish THg) = 0.211629 + −0.001341(Mean Percent Agriculture Cover), *F*_1,483_ = 18.89, *R*^2^ = 0.0354, *p* < 0.001) with the spatial distribution of agricultural land cover on the right

There are marked spatial patterns of fish Hg concentrations across New York State (Figs 11, 12). In general fish Hg concentrations are highest in the Adirondack and Catskill regions and the Hudson Highlands of eastern New York State (Millard et al. 2020b). Land cover is an important determinant of this spatial pattern of fish Hg concentrations, with higher concentrations under forest land cover and lower under agricultural land cover (Fig. 13). Forest cover coincides with attributes that facilitate elevated fish Hg concentrations, including abundant wetlands and waters characterized by low nutrients and productivity. Conversely, agricultural lands coincide with lower fish Hg concentrations, partly related to fewer wetlands. Denkenberger et al. (2020) found marked variation in the forms of Hg in rivers draining into Lake Ontario. They observed strong partitioning of inorganic Hg by suspended matter. Watersheds high in agricultural and urban land cover that supplied large quantities of suspended particulate matter tended to largely transport particulate forms of Hg, although this riverine response was muted by the presence of reservoirs. In contrast, in forested landscapes river Hg largely occurred in a dissolved form. Although land cover is an important determinate of fish Hg concentrations for areas with an abundance of forest cover, there is considerable variability in Hg concentrations in game fish (Figs 12, 13).

##### Avian piscivores

**Fig. 14:**
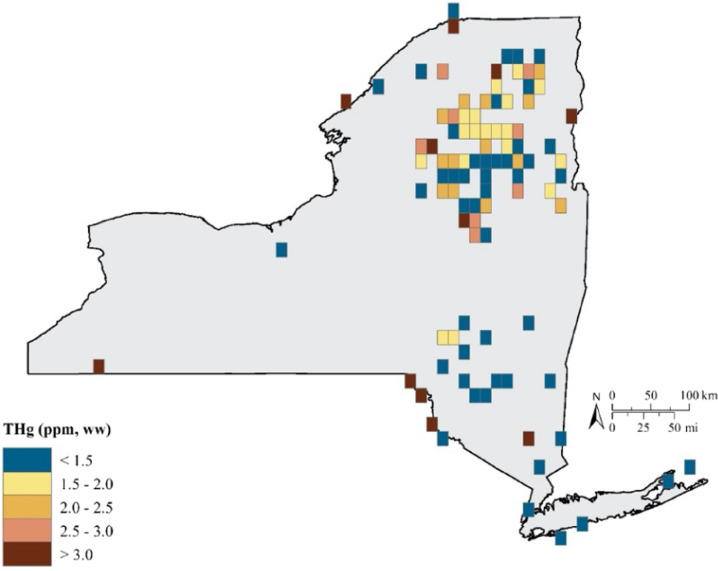
Avian piscivores sampled in New York State from 1970 through 2017. Of the grids sampled for avian piscivores (*n* = 97), 52 percent of the grids they occupy were above 1.5 ppm of total Hg in their blood (equivalent to a loss of 10% of reproductive success), while 33% were above 2.0 ppm or 20% loss, and 20% over 2.5 ppm or 30% loss (Table 5)

Birds that regularly forage on fish (piscivores) are often at risk from environmental Hg (Table 5). In New York State, larger avian piscivores (e.g., common loon, bald eagle, osprey) tend to have greater Hg body burdens than smaller avian piscivores (e.g., common merganser (*Mergus merganser*), belted kingfisher (*Megaceryle alcyon*), roseate tern *Sterna dougallii*). The greatest risk in New York State among these large piscivores is for common loons in the Adirondack Mountains (Schoch et al. 2020), and for bald eagles foraging in the Catskill Mountains and major rivers (e.g., Saint Lawrence, Hudson, Allegheny, and Susquehanna Rivers; DeSorbo et al. 2020; Fig. 14).

**Table 5:** Screening benchmarks for piscivorous and invertivorous birds (Evers 2018)

Avian Forage Guild of Interest	Screening benchmark: THg in blood (ppm, ww)	Endpoint of interest
Piscivores	<1.5	No observed effect level
	1.5–2.0	10% fewer fledged young
	2.0–2.5	20% fewer fledged young
	2.5–3.0	30% fewer fledged young
	>3.0	≥40% fewer fledged young
Invertivores	<0.40	No observed effect level
	0.40–0.70	<10% fewer fledged young
	0.70–1.2	10% fewer fledged young
	1.2–1.7	20% fewer fledged young
	>1.7	≥30% fewer fledged young

##### Avian invertivores

**Fig. 15:**
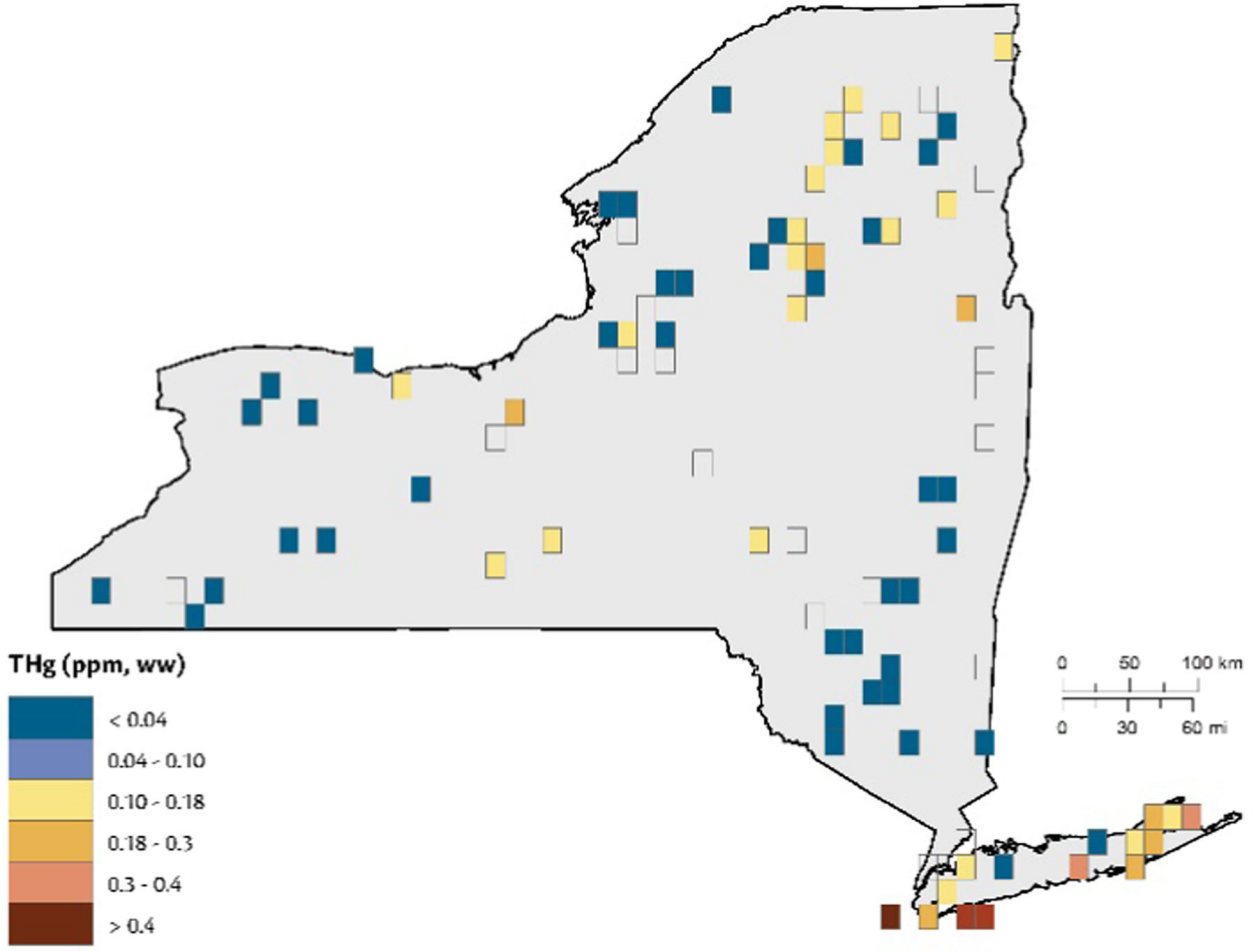
From 1970 to 2017, a total of 93 grids (representing 9% coverage) have been sampled for avian invertivores. Target songbirds were found in 73 of these grids, and 44% of these birds had >0.4 ppm total blood Hg; 17% were >0.7 ppm; 7% were >1.2 ppm; and 4% were >1.7 ppm (Table 5)

Methylmercury from aquatic ecosystems can enter terrestrial food webs via predatory invertebrates, such as dragonflies and spiders (Seewagen et al. 2016; Nelson et al. 2020; Sauer et al. 2020a). Consumers of these and other invertebrates include avian invertivores, which have been identified to be of concern due to MeHg exposure (Evers et al. 2005; Jackson et al. 2011, 2015; Cristol and Evers 2020). The highest Hg concentrations and associated health risk in the state are for the following species: saltmarsh and seaside sparrows (*Ammospiza caudacuta* and *A. maritima*, respectively) in the estuaries of Long Island; palm warbler (*Setophaga palmarum*) and rusty blackbird (*Euphagus carolinus*) in the Adirondack Mountains; swamp sparrow (*Melospiza georgiana*) and red-winged blackbird (*Agelaius phoeniceus*) in the Catskill Mountains; and marsh wren (*Cistothorus palustris*) in the Montezuma Lakes complex (Fig. 15). Mercury body burdens for these species are at levels that may cause adverse reproductive impacts at a scale of concern.

### Habitats sensitive to mercury inputs

Habitats where relatively small inputs of Hg can be transported, methylated and bioaccumulate to create areas of concern for fish, wildlife, and people are known as biological Hg hotspots (Evers et al. 2007). Mercury sensitive habitats identified in New York State based on observed Hg concentrations in biota include:

1. Estuaries: Mercury concentrations in blood and feather tissue from the saltmarsh sparrow on Long Island regularly exceeds levels that cause lower reproductive success in songbirds (Lane et al. 2020). Saltmarsh and seaside sparrows are at particular risk because they often feed on spiders—a high MeHg food source (Cristol et al. 2008).
2. Sphagnum bogs and other wetlands: Wetlands are environments with efficient conversion of inorganic Hg to MeHg (Wang et al. 2020). In the Adirondack Park and elsewhere in northern New York State, sphagnum bogs generate elevated levels of MeHg. The transfer of MeHg within and between aquatic and terrestrial food webs are important pathways for biomagnification and create risk for some avian invertivores such as the palm warbler (*Setophaga palmarum*) and rusty blackbird (Edmonds et al. 2010; Perkins et al. 2020; Sauer et al. 2020a).
3. High-elevation boreal forests: Montane habitats generally receive higher rates of atmospheric Hg deposition due to increased precipitation quantity and cloud cover (Blackwell et al. 2014; Gerson et al. 2017). An elevational gradient of increasing Hg exposure was found in songbirds including Swainson’s (*Catharus ustulatus*), hermit (*C. guttatus*), and Bicknell’s (*C. bicknelli*) thrush on Whiteface Mountain in the Adirondack Park—although the risk to reproductive success is minor (Rimmer et al. 2005, 2020; Sauer et al. 2020b).
4. Streams and rivers in forested watersheds: Based on macroinvertebrates (e.g., odonate larvae) and fish, watersheds with higher forest cover and wetlands have higher MeHg concentrations that frequently exceed fish-related guidelines for human health and wildlife (Riva-Murray et al. 2020a, 2020b; Millard et al. 2020b). The belted kingfisher (*Megaceryle alcyon)* and Louisiana waterthrush (*Parkesia motacilla)* are good bioindicators for such systems (Evers et al. 2005; Jackson et al. 2011).
5. Acidic lakes with wetland shorelines in forested watersheds: While most lakes have the potential for high trophic level biota with elevated Hg body burdens, oligotrophic lakes with a pH below 6.3, with significant shoreline wetlands, and/or fluctuating water levels are of greatest concern for piscivorous species such as game fish (Millard et al. 2020b), the common loon (Schoch et al. 2020) and bald eagle (DeSorbo et al. 2020).

The Northeastern Highlands Ecoregion (particularly the Adirondack and Catskill regions) of New York State are particularly sensitive to Hg pollution. The impact of Hg emissions and deposition is exacerbated by watershed, stream, and lake characteristics in areas with abundant forests and wetlands—areas that enhance Hg inputs, transport, methylation, and uptake to elevate concentrations in aquatic food webs resulting in elevated Hg concentrations in game fish and avian piscivores and invertivores. These regions have also been impacted by historical acid deposition.

#### Long-term changes in mercury in biota

Multiple bioindicators are needed to confidently track changes. For some biota, such as songbirds, long-term trends in Hg exposure can be tracked with museum specimens and compared with recent sampling (Dzielski et al. 2020; Perkins et al. 2020). There is strong evidence that long-term increases in environmental Hg loads (Drevnick et al. 2012) and subsequent effects have increased significantly since the mid-1800s (Perkins et al. 2020), but linking Hg body burdens of biota in New York State with Hg emission patterns remains challenging (Dzielski et al. 2020).

**Fig. 16:**
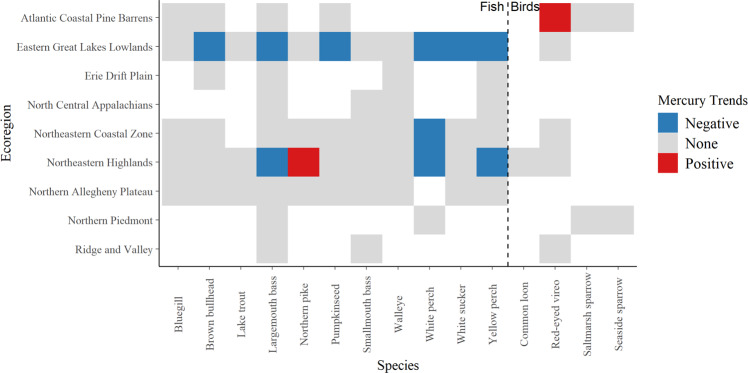
Long-term trends in Hg concentrations on fish and birds in ecoregions of New York State. A blue square indicates a significant decreasing trend for a given species, a red square a significant increasing trend, and a gray square no significant trend

Time series analysis revealed limited significant trends in Hg concentrations in fish or birds for most ecoregions of New York State (Fig. 16). The most consistent significant decreasing trends were evident across multiple fish species in the Eastern Great Lakes Lowlands (Fig. 16). These trends may reflect decreases in releases of Hg to the Great Lakes region coupled with decreases in atmospheric Hg deposition. Ye et al. (2020) found that lands immediately adjacent to the Great Lakes had among the highest atmospheric Hg deposition in New York State (Fig. 6). To a lesser extent decreasing trends were also evident for largemouth bass, white perch and yellow perch in the Northern Highlands. These decreasing trends are tempered by increasing concentrations of Hg in northern pike (*Esox lucius*; Fig. 16). Trends in fish Hg concentrations that are inconsistent with those in atmospheric Hg deposition are quite common and can result from the influence of many factors including response lags, climate variation, and shifts in food sources (Wang et al. 2019).

Game fish have been monitored for Hg in the Great Lakes adjacent to New York State for almost 50 years (Richter and Skinner 2020; Fig. 16). Over this period, many environmental factors influencing Hg bioavailability have changed, including Hg deposition rates, climate, hydrology and lake stage, surrounding terrestrial habitat, and food webs. Using fillet concentrations, Hg exposure levels were tracked in 16 fish species over a 40-year period to determine annual changes in fish exposure risk and human risk from consumption (Richter and Skinner 2020). The steepest declines in Hg concentrations occurred from the 1970s–80s for yellow perch (*n* = 999) and largemouth bass (*n* = 1344). From the 1980s onward, fish Hg concentrations have continued to generally decline. Zhou et al. (2017) also found decreasing Hg trends (in lake trout, *Salvelinus namaycush*, and walleye) across the Great Lakes for the past four decades, except in the past decade where there were increases for Lake Erie and Lake Ontario.

Common loons have been monitored for Hg exposure in the Adirondack Park since 1998 (Schoch et al. 2020) and optimal sampling strategies are now identified, although lake acidity (Yang et al. 2020) and likely other abiotic factors (Buxton et al. 2020) need to be considered when evaluating trends. Mercury can be elevated in the fish that loons consume, which may adversely affect loon populations. As the top predator in montane lake ecosystems, loons show Hg concentrations that are indicative of risk throughout their breeding range in New York State. Using Hg in blood and egg samples from breeding loons in 116 lakes throughout Adirondack Park from 1998 to 2016, Schoch et al. (2020) tracked MeHg availability and evaluated risk to the common loon (Fig. 16). Concentrations of Hg in breeding loons increased 5.7 percent per year from 1998 to 2010, and then stabilized from 2010 to 2016. Recovery of Hg concentrations in loons has been delayed compared to trends in local atmospheric Hg deposition (Fig. 8), even though there has been a long-term recovery of lakes in the Adirondack region to decreases in acidic deposition (Waller et al. 2012; Driscoll et al. 2016).

Mercury exposure high enough to cause reproductive impacts has been identified in saltmarsh sparrows (Lane et al. 2011, 2020), a species classified as endangered on the International Union for Conservation of Nature (IUCN) Red List. Since 2000, monitoring projects have tracked Hg concentrations in saltmarsh sparrow blood to understand how the risk of Hg effects has changed over time across Long Island (Lane et al. 2020; Fig. 16). Risk of Hg exposure is declining, but remains elevated across New York State marshes, and there is considerable variation in blood Hg concentrations within the breeding season. Concentrations peak in mid-July and such seasonal patterns need to be considered for long-term monitoring. Most marshes have cyclical patterns of annual Hg exposure, which may relate to annual variation in local Hg deposition, tidal marsh flooding, and variation in food availability.

For fish species, more than 50 years of Hg data are available, and are especially robust for yellow perch and largemouth bass, which show statistically significant declines statewide based on the NYSMD and for the Eastern Great Lakes Lowlands, specifically, the slope parameters are significant for yellow perch (*β* = −0.022 [−0.033, −0.011]) and largemouth bass (*β* = −0.015 [−0.030, 0.000]). For birds, the temporal span of data varies from six to more than 20 years among species. The red-eyed vireo (*Vireo olivaceus*) sampled in Long Island’s forests shows an increasing trend (slope parameter *β* = 0.26, 95% credible interval = [0.03, 0.49]), yet in nearby estuaries, the saltmarsh sparrow shows a declining trend (slope parameter *β* = −0.055, 95% credible interval = [−0.144, 0.036]). Ultimately, ecoregions may respond differently to changes in environmental Hg loads, as will taxa. Therefore, use of fish and bird bioindicators for multiple ecoregions is essential to understand and track the availability of MeHg across New York State.

Mercury concentrations in biota of New York State have declined or remained constant over the last four decades, concurrent with decreased air emissions from regional and U.S. sources (Figs. 4, 5, 7, 8). After initial declines, however, concentrations of Hg in some fishes and birds from certain locations have increased in recent years, revealing the complexities of trajectories of Hg recovery (Zhou et al. 2017). Because there is not a demonstrated relationship between atmospheric Hg deposition and biotic uptake (Rimmer et al. 2020; Dzielski et al. 2020), Hg monitoring in biota is of ongoing importance.

#### Long-term influences from climate change

The results of these studies suggest that climate change could potentially affect future Hg deposition. For example, climate predictions for the northeastern United States anticipate increased precipitation amounts and increased temperature, which may in turn drive increases in Hg deposition. This projection underscores the importance of further reduction of anthropogenic Hg emissions in order to address the potential influence climate change may have on Hg exposure.

Factors affecting Hg transport, transformations, and bioaccumulation include: (1) increases in rainfall amount and variability, which create conditions of increased transport and leaching from soil and enhanced wet-dry cycles that augment methylation (Burns et al. 2014a; Watras et al. 2020; Wang et al. 2020); (2) enhanced growth of trees which is a major pathway for atmospheric Hg deposition (Blackwell et al. 2014; Jiskra et al. 2018); (3) higher water temperatures which are associated with higher Hg methylation rates and fish Hg concentrations, and strengthen and extend the duration of thermal stratification in lakes which may increase depletion of dissolved oxygen and methylation of Hg (Bodaly et al. 1993; Warren et al. 2016); and (4) increases in water temperature and fluctuating water tables in stream water enhancing MeHg concentrations (Burns et al. 2014b).

Recent evidence suggests that climate-related factors influence patterns of Hg exposure in aquatic and terrestrial biota. Increasing temperature and rainfall, combined with shifting habitat distributions, will shape future Hg deposition, transport, and methylation, and in turn, exposure risk. Understanding how these large-scale factors will play out on smaller spatial and temporal scales will require continued research and monitoring (Swinton and Nierzwicki-Bauer 2020; Adams et al. 2020).

### What are key mercury policy connections in New York State and beyond?

While the timing and magnitude of the response will vary, further controls on Hg emission sources are expected to lower Hg concentrations in food webs, yielding multiple benefits to fish, wildlife, and people in New York State and the surrounding region. Improvements are anticipated to be greatest for inland surface waters and will be roughly proportional to declines in Hg deposition (Knightes et al. 2009), which most closely track trends in regional and U.S. air emissions. This scientific synthesis of Hg in air, water, invertebrates, fish, and birds characterizes the status and effects of Hg pollution across New York State. Information from existing data and Hg monitoring programs can inform many of the regional, national, and global policy initiatives currently implemented or in process.

#### U.S. mercury emissions

Mercury pollution in the U.S. is regulated by an array of state and federal regulations (see: http://www.epa.gov/hg/). There has been substantial progress in regulatory efforts to decrease Hg emissions from major source categories. Specifically, the USEPA has: (1) finalized maximum standards for Hg from coal-fired power plants; (2) created national emissions standards for hazardous air pollutants for gold ore processing and production facilities; (3) finalized rules to control Hg emissions from Portland cement manufacturing facilities; and (4) proposed new source performance standards and emissions guidelines for new and existing sewage sludge incinerators. Mercury reductions from electric utilities have also occurred indirectly through controls on emissions of sulfur dioxide and nitrogen oxides (Zhang et al. 2016).

The MATS rule has resulted in marked decreases in Hg emissions from electric utilities (Fig. 4). This rule is integral to meeting U.S. commitments under the international Minamata Convention on Mercury. In 2020, the USEPA decided to rescind the finding that regulatory Hg is “appropriate and necessary”, a modification that has led to challenges to the MATS rule and its potential repeal.

Many states have implemented their own regulations to limit Hg emissions, which have often been more stringent than federal regulations (Rallo et al. 2012). One novel approach is use of provisions of the Clean Water Act and establishing a total maximum daily load (TMDL) for Hg that can have an indirect effect on emissions (https://neiwpcc.org/our-programs/nps/mercury/mercury-tmdl/). New York State and the six New England states began implementation of the Northeast Regional Mercury Total Maximum Daily Load in 2007 to reduce fish Hg concentrations in all surface water bodies to less than 0.3 ppm. The TMDL effort has harnessed and focused efforts within the Northeast to measure and identify impaired water bodies and to reduce Hg use and emissions within the region but has encountered some hurdles as well because of limited ability of the states to affect Hg emissions outside their region (Selin 2011).

#### Minamata Convention on Mercury

The Minamata Convention on Mercury is a global treaty intended to protect human health and the environment from anthropogenic emissions and releases of Hg. In 2013 the text of the Convention was approved by delegates representing nearly 140 countries. The Convention was adopted and signed in 2013 at the Diplomatic Conference in Kumamoto, Japan. The Convention entered into force in 2017. More than 120 countries have ratified or accessioned the Convention, including the United States (see https://www.mercuryconvention.org/). Pursuant to Article 23 of the Minamata Convention, a governing body called the Conference of the Parties (COP) was established. The COP is responsible for advancing implementation of the Convention and for keeping the Convention under continuous review and evaluation. To evaluate the effectiveness of the Minamata Convention’s success, Hg monitoring efforts at the global level are planned (Evers and Sunderland 2019). Decisions relevant to these responsibilities are made during meetings of the COP. The Minamata Convention is expected to provide substantial benefits to the U.S. in excess of $100 billion as cumulative lifetime health benefits as well as economic benefits, primarily due to improved health outcomes from consumption of marine fish (Giang and Selin 2016). Residents of New York are expected to realize substantial benefits from implementation of the Minamata Convention due to anticipated decreases in atmospheric Hg deposition from global sources (Lin et al. 2012), as well because of state’s abundant coastal marine waters and previously identified high levels of marine fish consumption and associated high Hg blood levels in coastal areas (Mahaffey et al. 2009).

#### Science informs policy—a mercury policy timeline

There has been substantial progress in regulating Hg use, emissions, and releases over the past four decades. The scientific understanding of the harm to human health and the environment from Hg increased in the 1980s and 1990s, when U.S. policies to control Hg trade, its use in products and industry, and its release in waste streams began to be instilled at state and regional levels (e.g., the Great Lakes and New England).

In parallel, local policymaking and national assessment of Hg effects were conducted that would eventually result in the MATS rule. Efforts to monetize the benefits of controls on Hg releases have found that the direct health benefits of controls are sizable (Giang and Selin 2016). The MATS rule was important in establishing a science-based policy for the United States to participate in a leadership role for creating the Minamata Convention on Mercury. Tracking Hg in the environment and its impact on human health is an important next step in the control of Hg use, emissions, and releases. The relationships among key compartments such as air, biota, and humans are complex and often not linear.

The complexity and interactions of the drivers behind the transport of Hg, methylation, and subsequent transfer through the food web requires monitoring to understand and evaluate the success of the MATS rule and the Minamata Convention. Tracking Hg over time also requires carefully designed sampling approaches that can generate standardized and comparable data over time. Preliminary approaches are being generated for the Minamata Convention (e.g., biota; Evers and Sunderland 2019) and with an expectation of initiation in the near future.
